# Supplementary figures and images for: Micrococcal nuclease sequencing of porcine sperm suggests enriched co-location between retained histones and genomic regions related to semen quality and early embryo development
Source: PeerJ. 2023 Jun 21;11:e15520. doi: 10.7717/peerj.15520 (PMC10290446; doi:10.7717/peerj.15520)

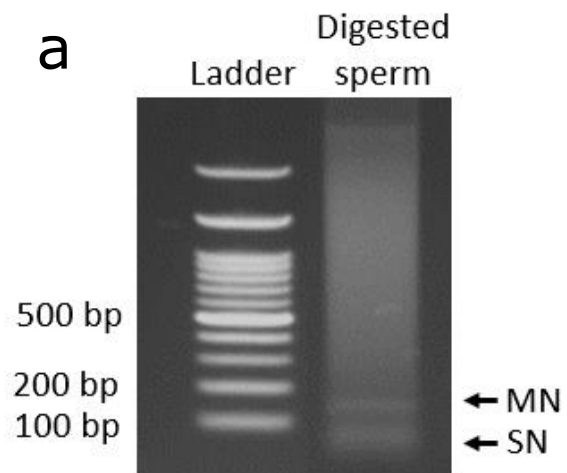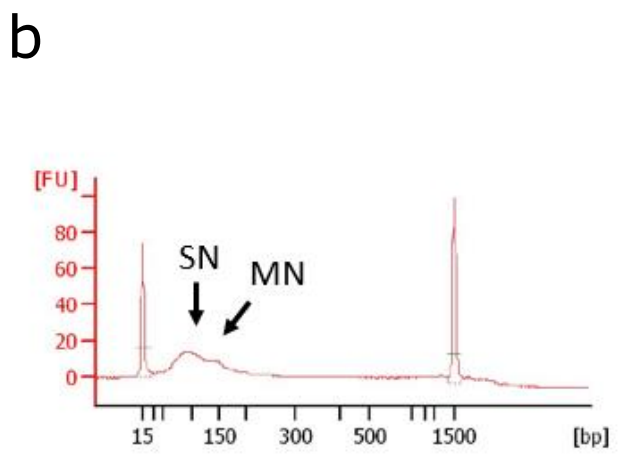

Supplement: Supplemental Information 1 — a. Agarose gel electrophoresis of the sperm chromatin after Micrococcal nuclease digestion resulted in one mono-nucleosomal (MN) (~ 147 bp) and one sub-nucleosomal (SN) (<100 bp) bands. Left: 100 bp DNA ladder; Right: ~ 300 ng of MNase digested sperm chromatin. b. Bioanalyzer electropherogram profile of a MNase digested sample showing the SN and MN DNA fractions. x-axis: bp fragment length; y-axis: FU (signal intensity) of the DNA fragments. [file peerj-11-15520-s001.pdf]

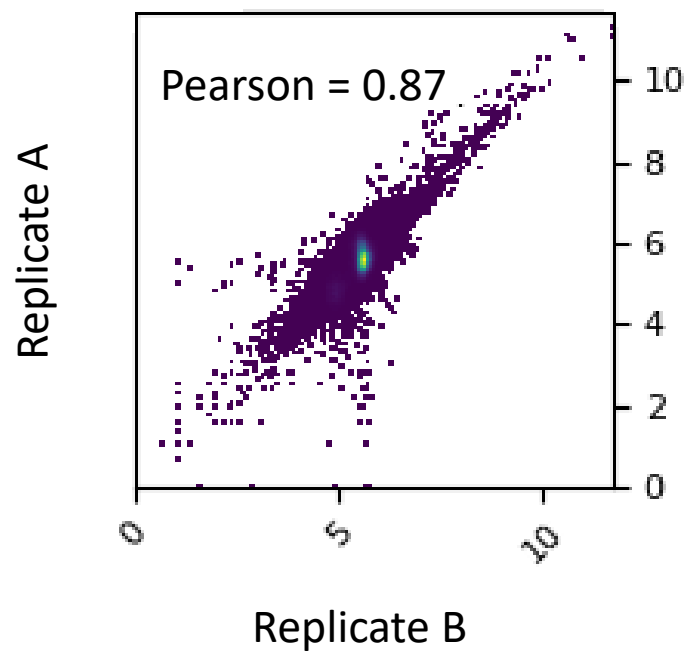

Supplement: Supplemental Information 2 — Scatterplots showing the Pearson’s correlation of the normalized MNase-Seq signals between the two biological replicates. [file peerj-11-15520-s002.pdf]

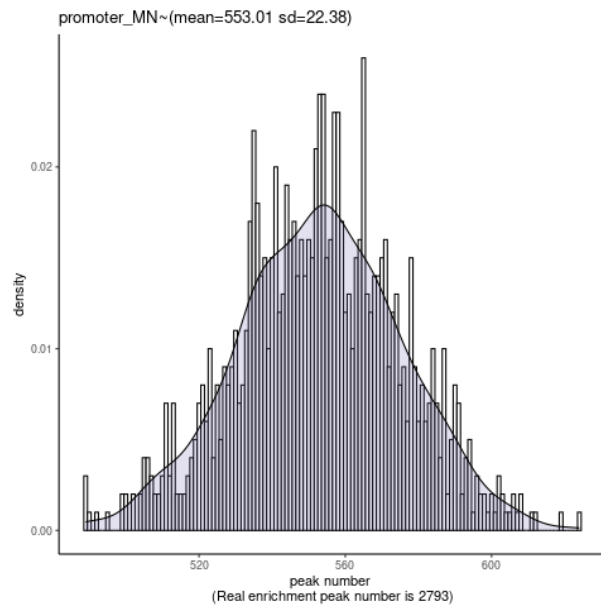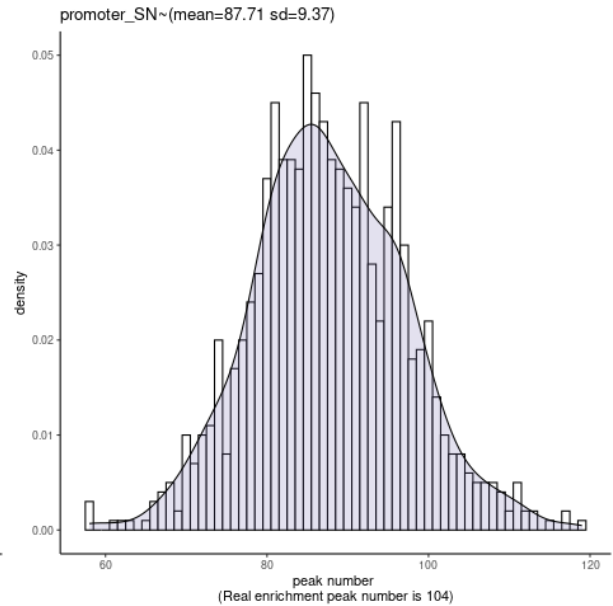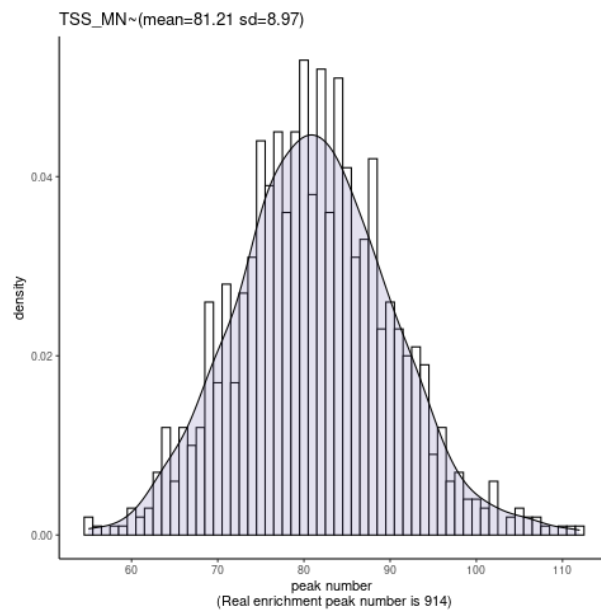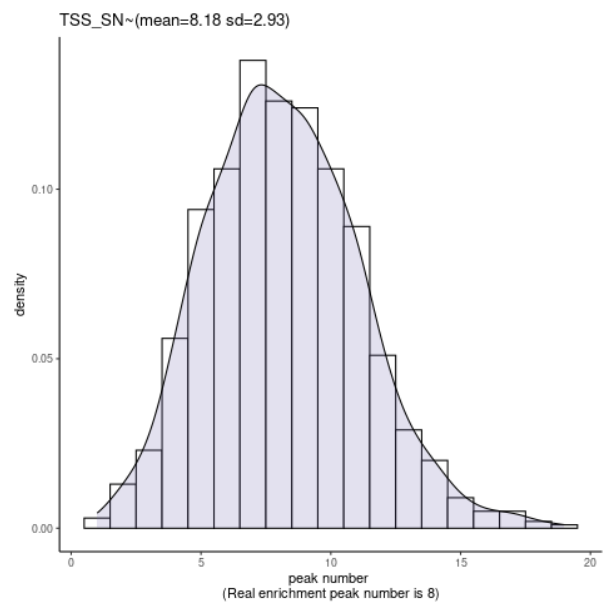

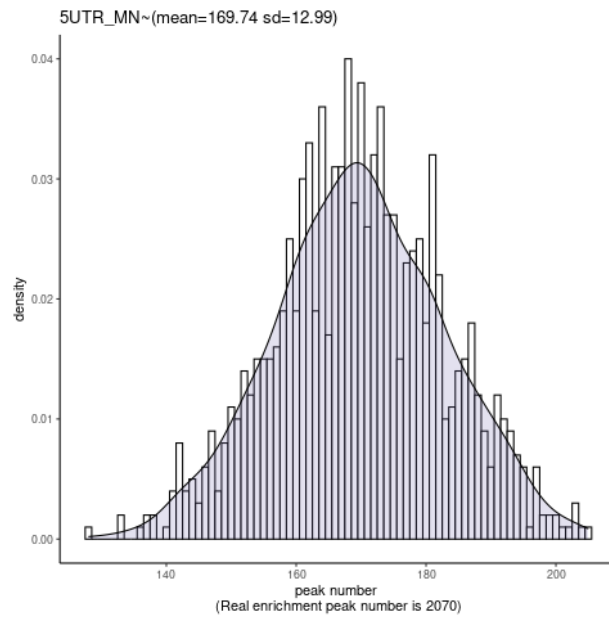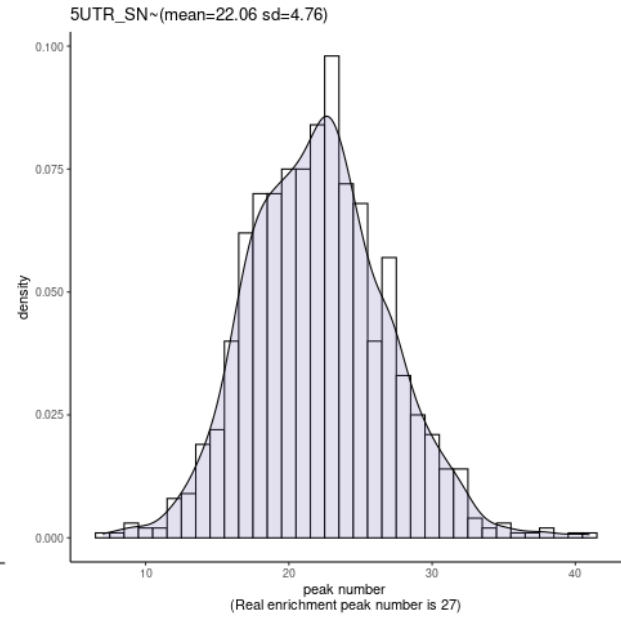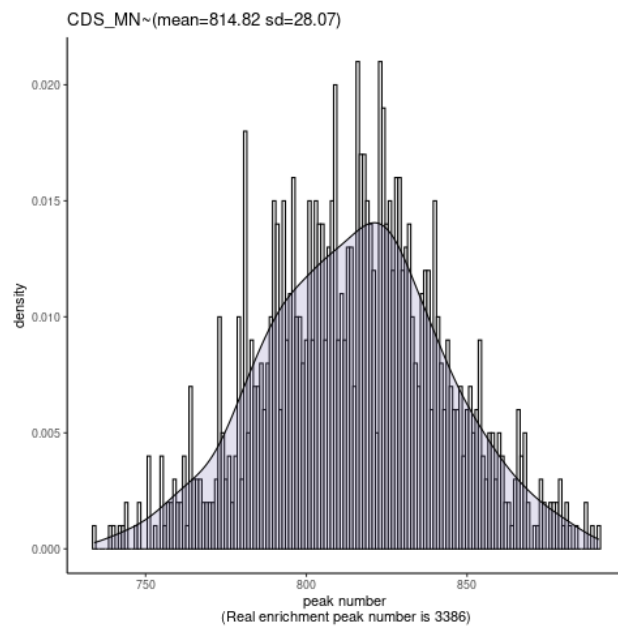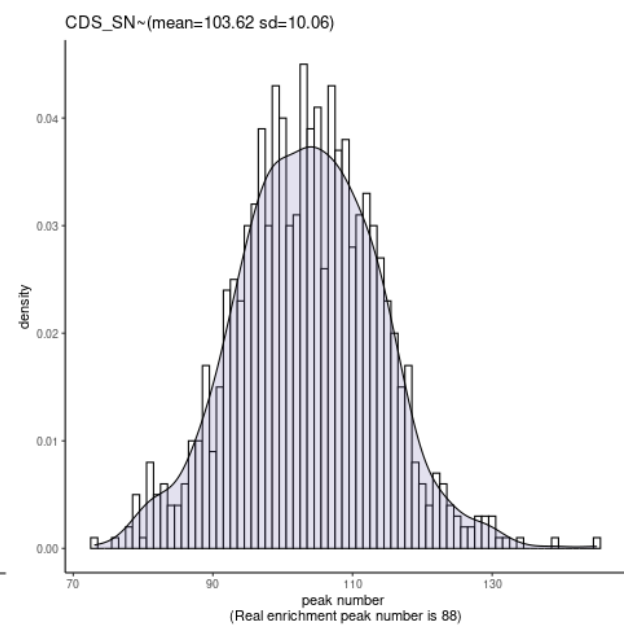

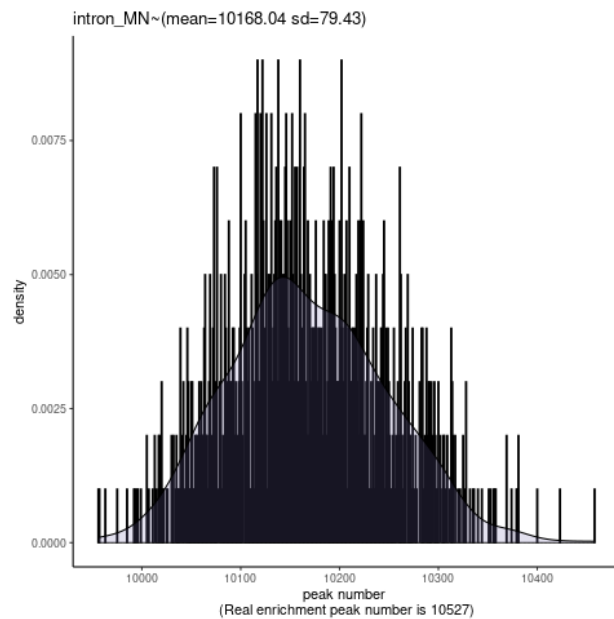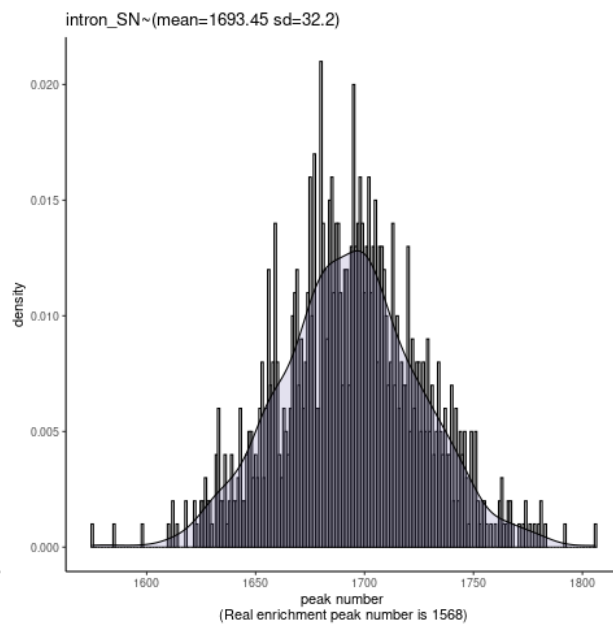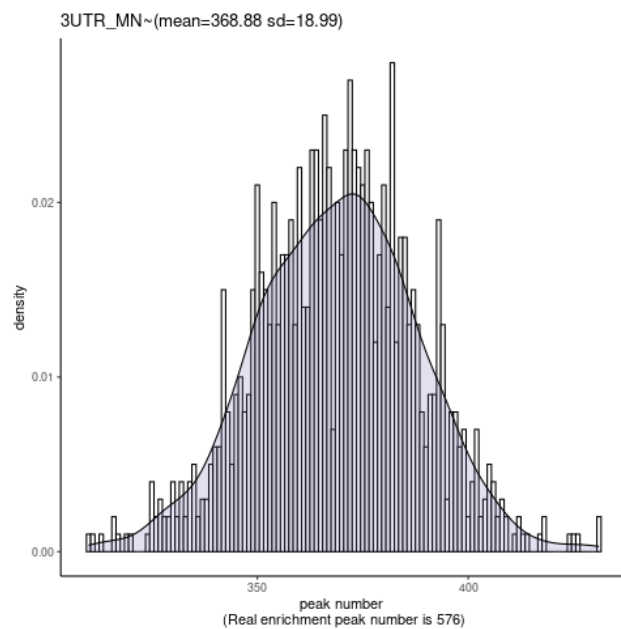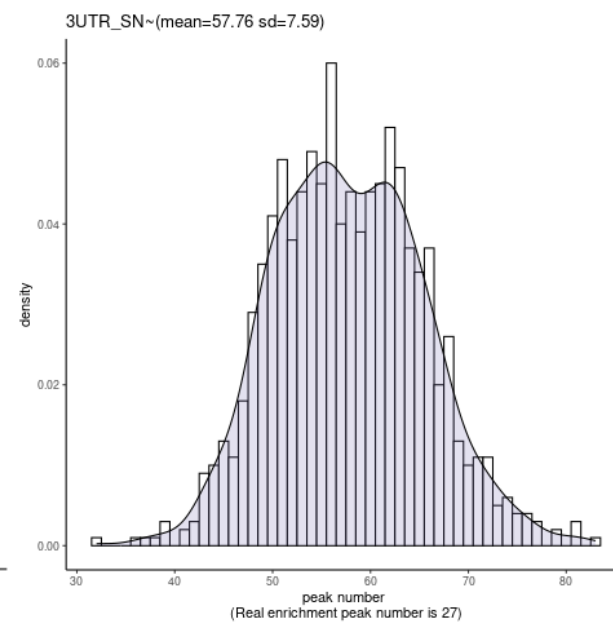

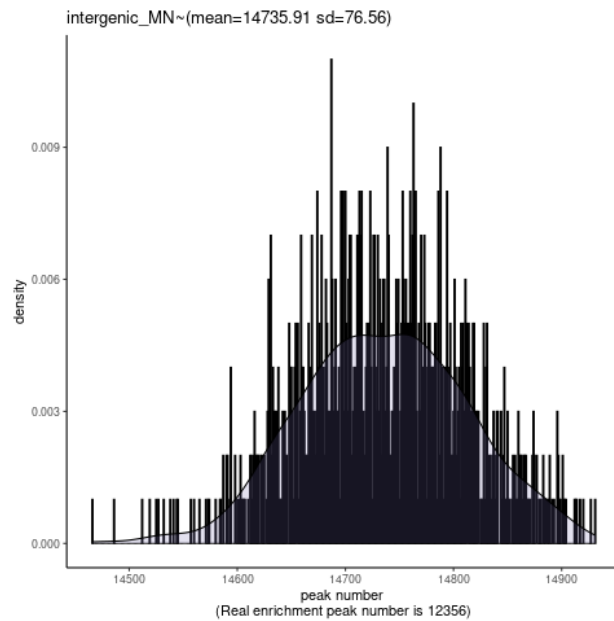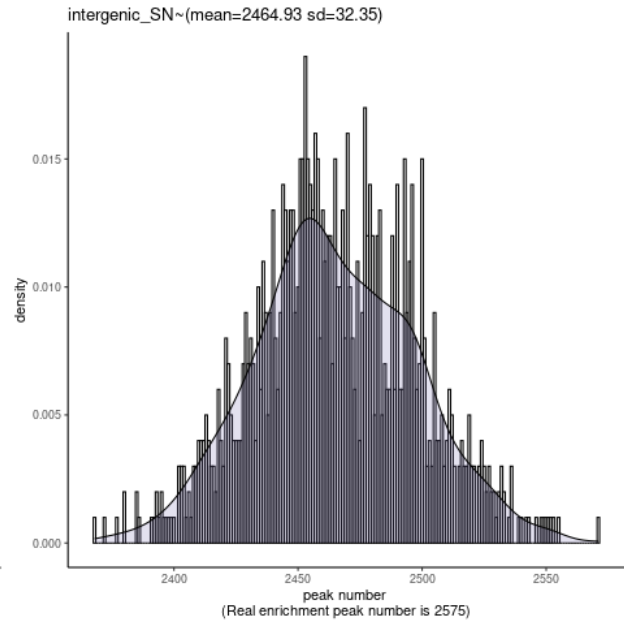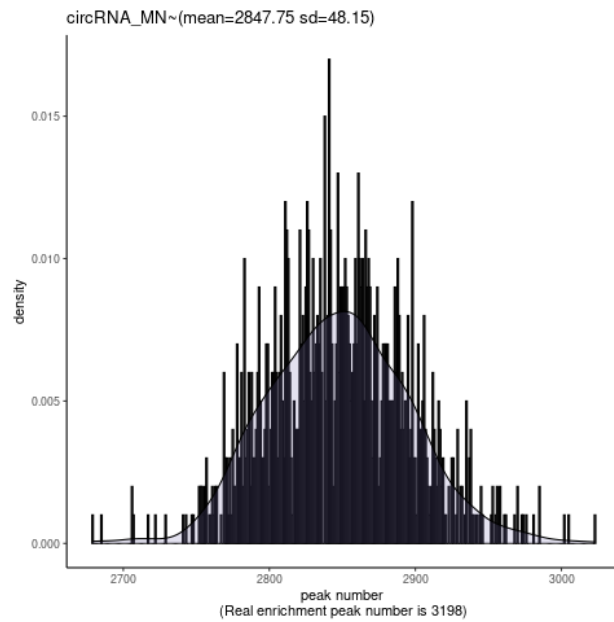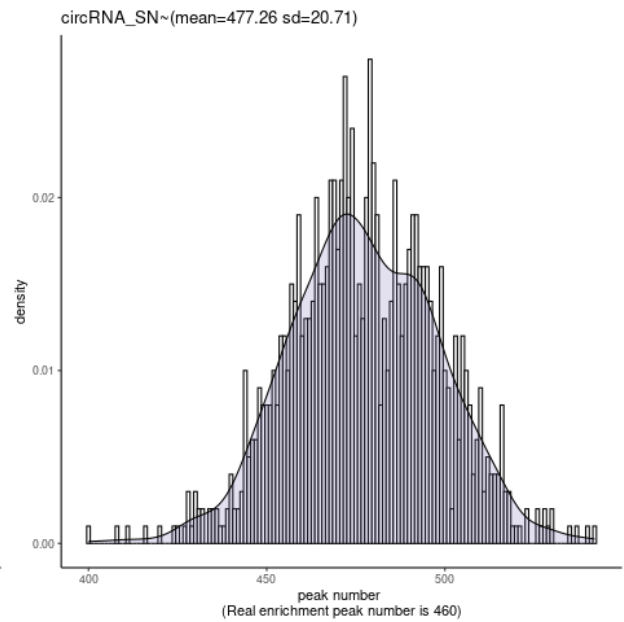

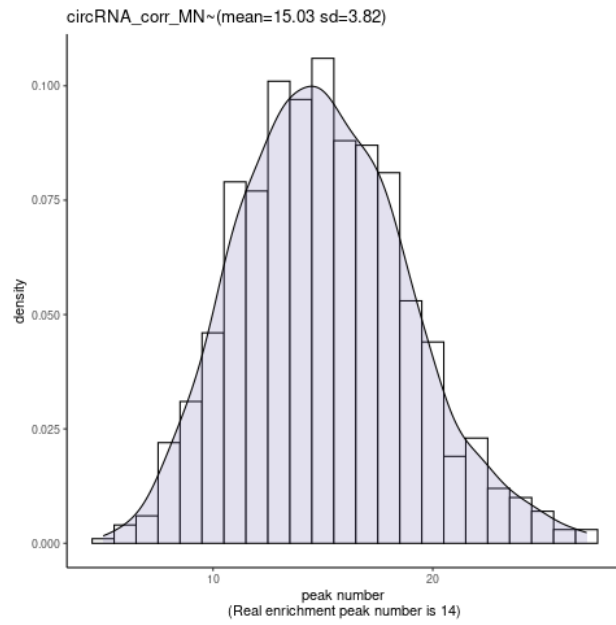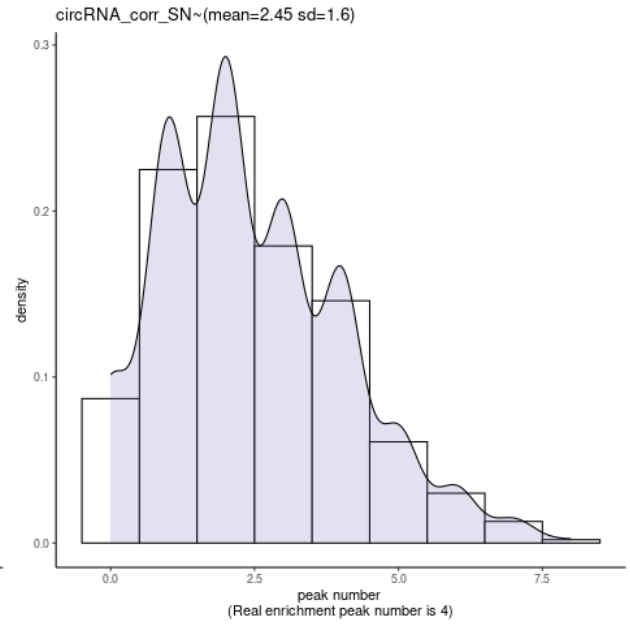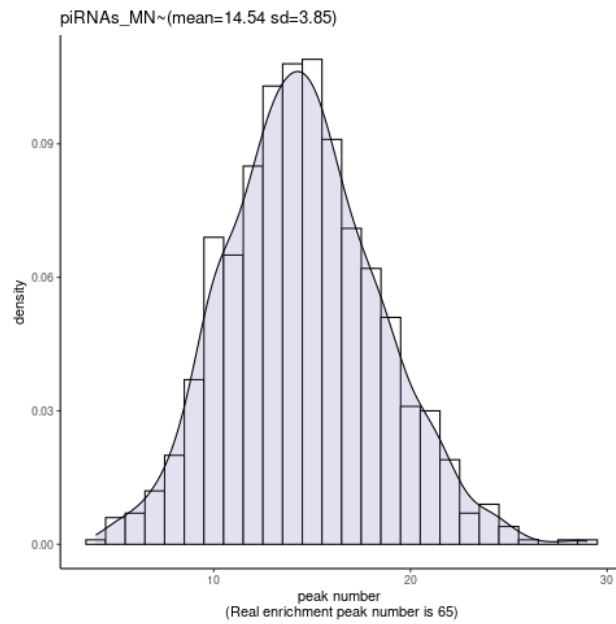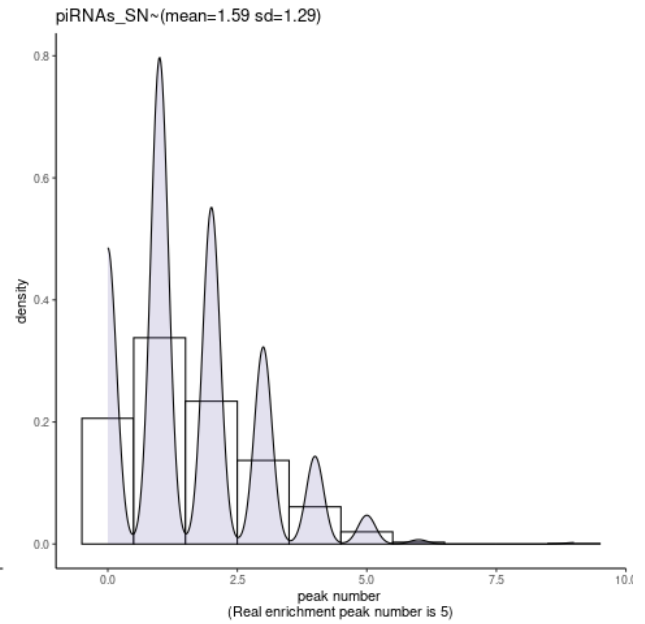

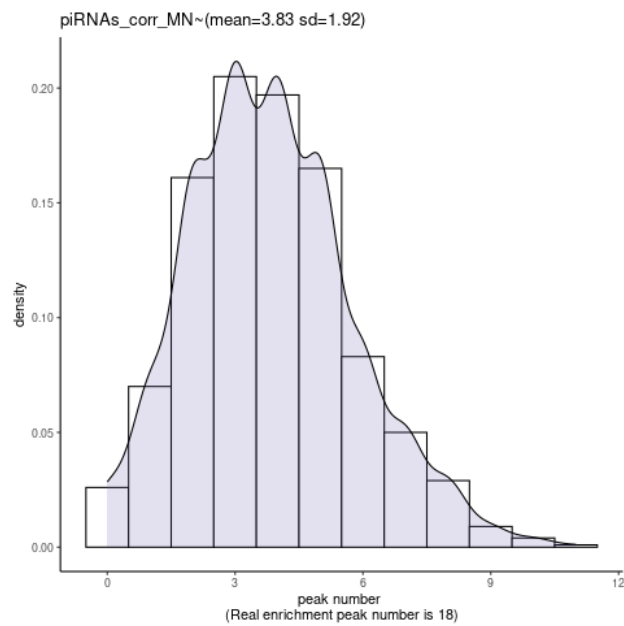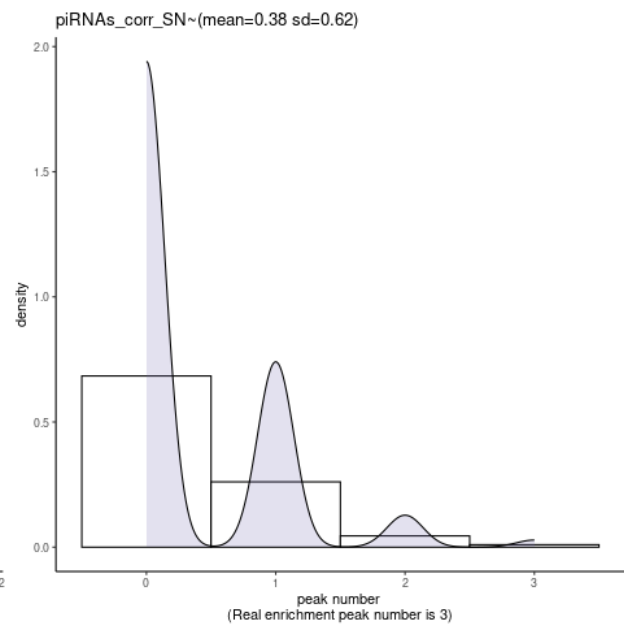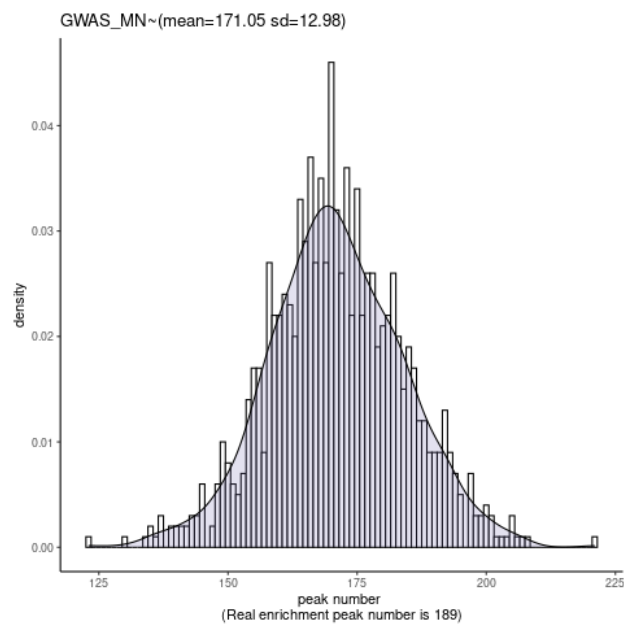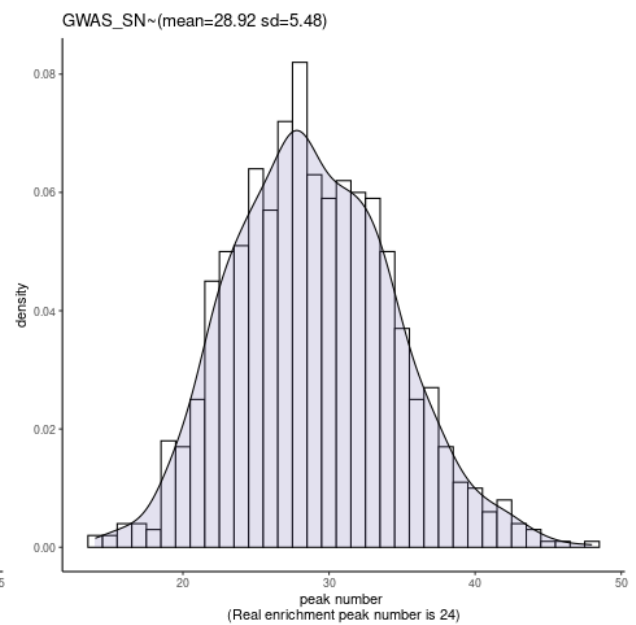

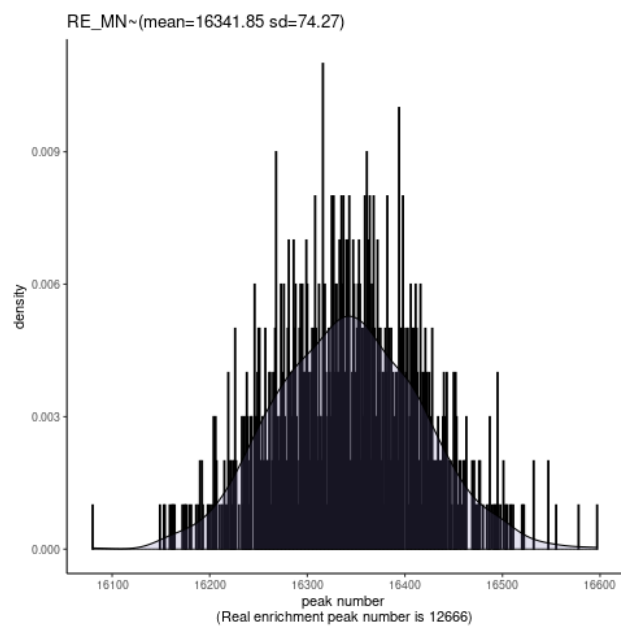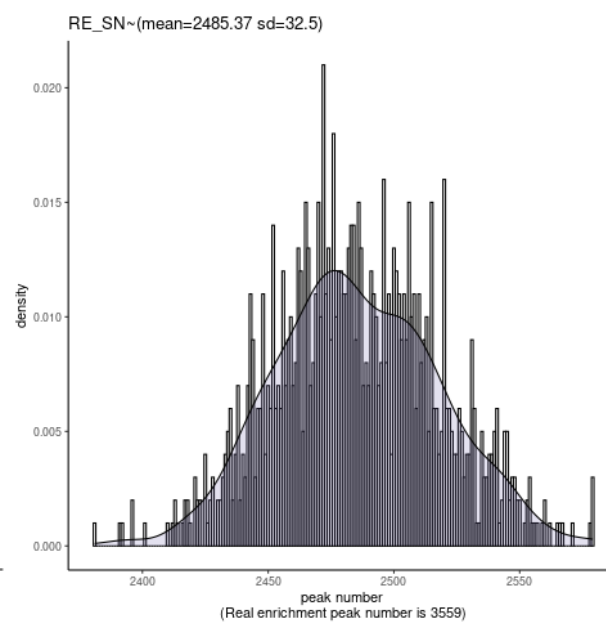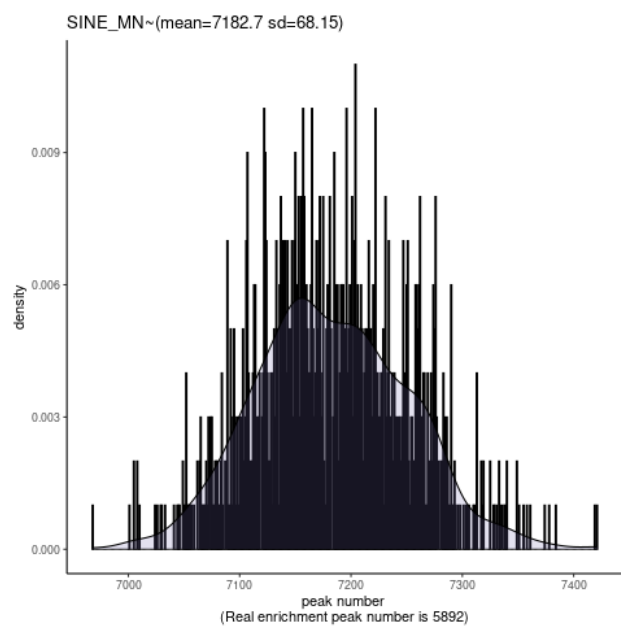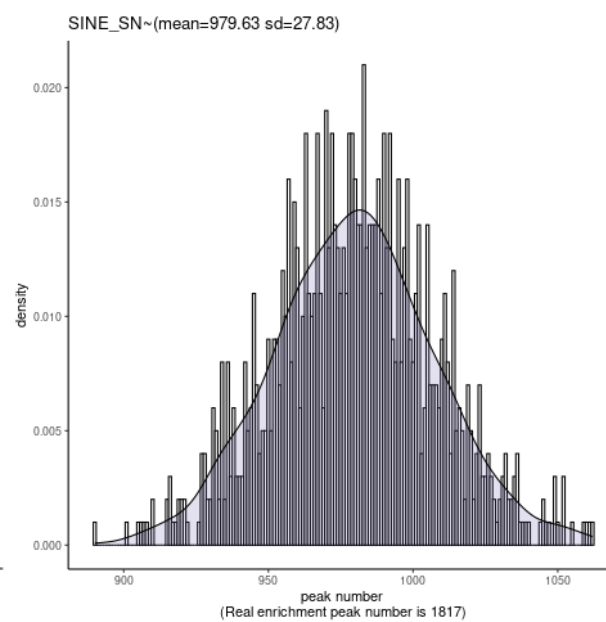

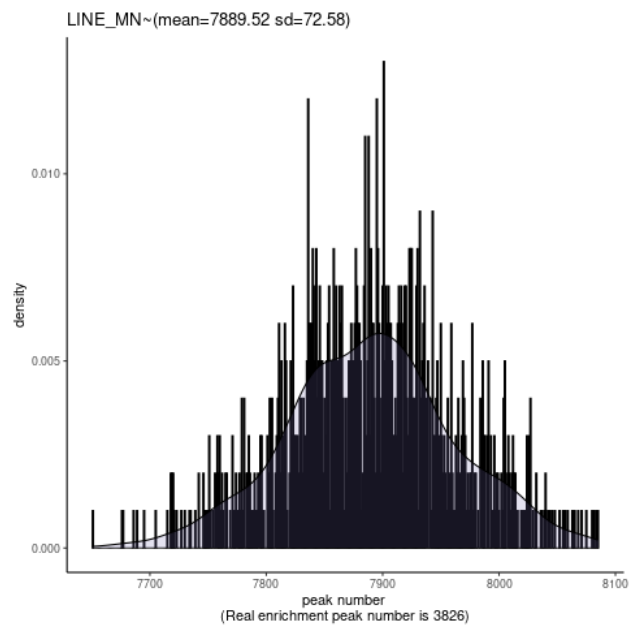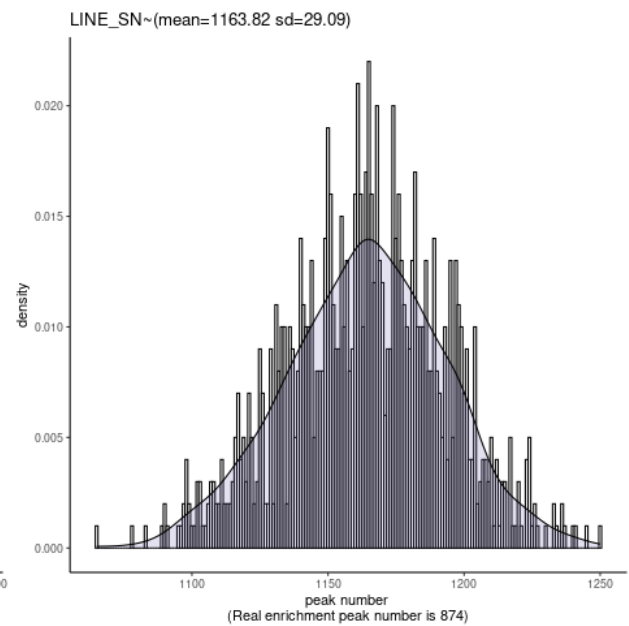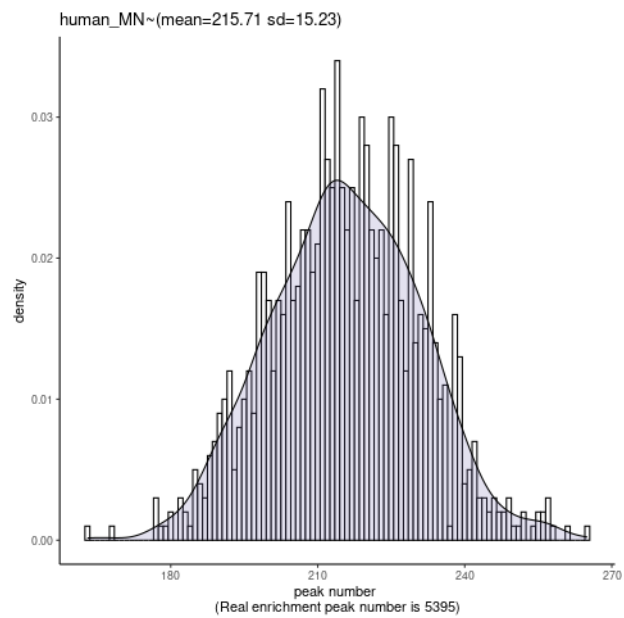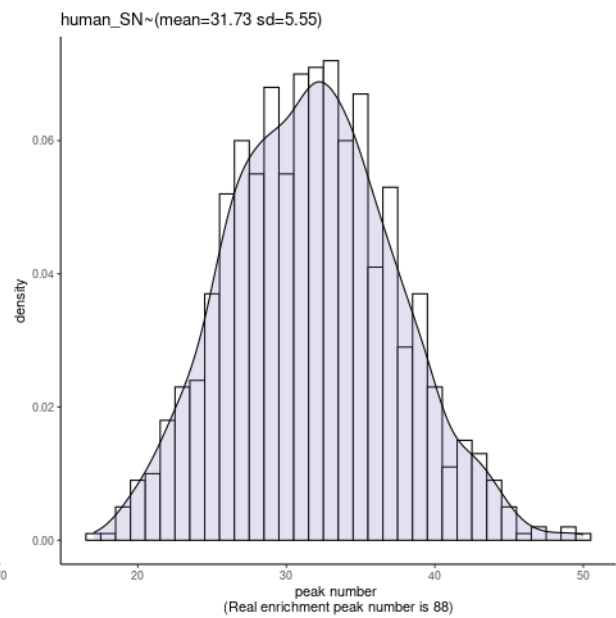

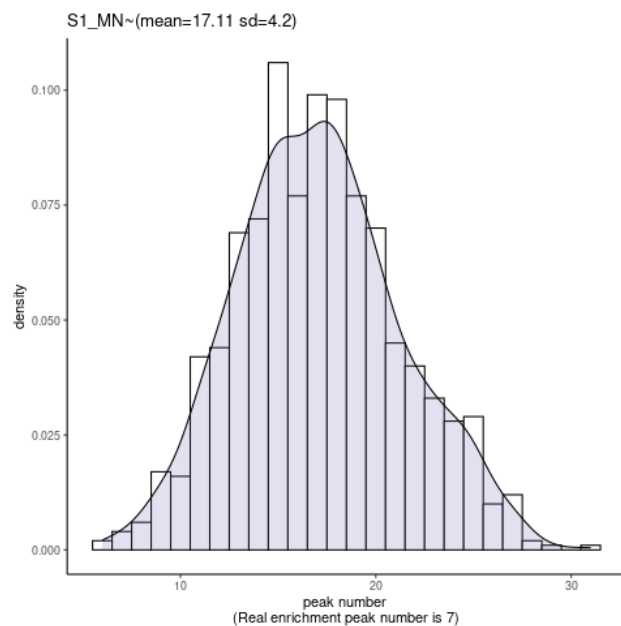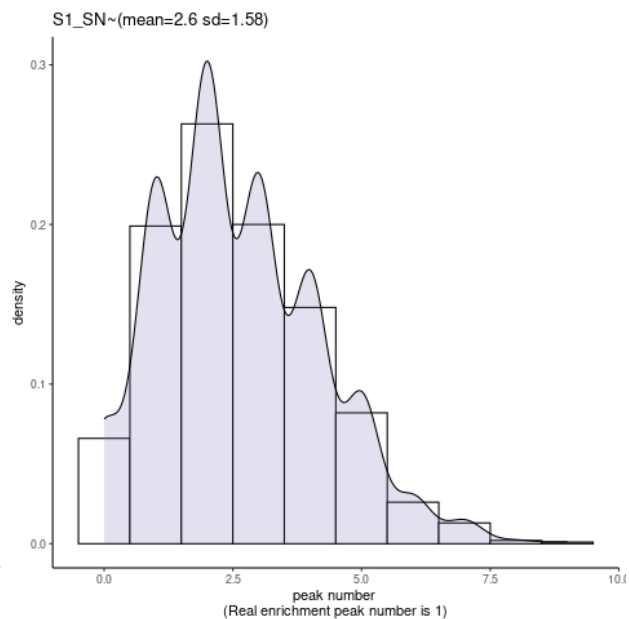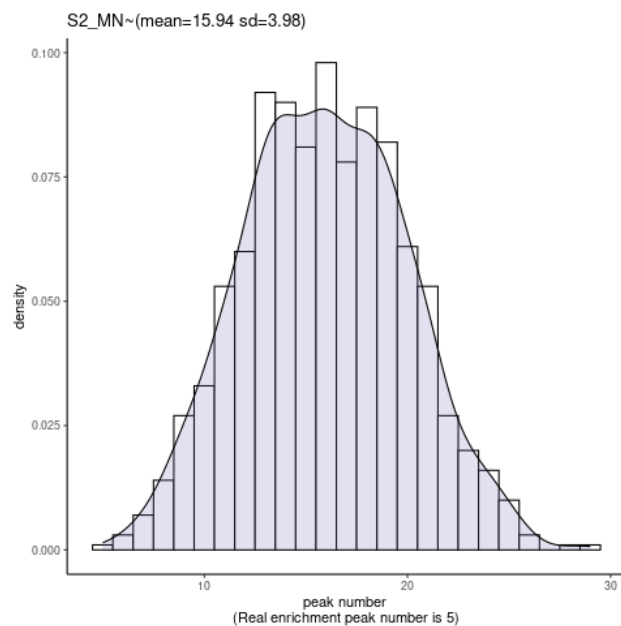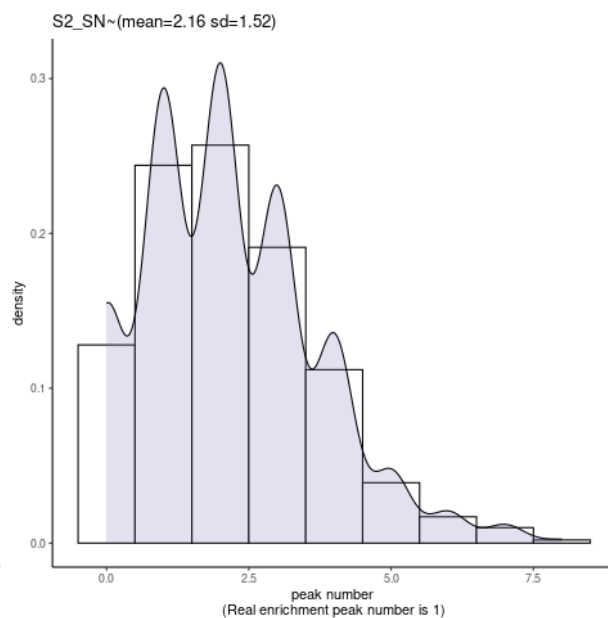

Supplement: Supplemental Information 3 [file peerj-11-15520-s003.pdf]
